# Increased levels of a subset of angiogenesis-related plasma proteins in essential thrombocythemia

**DOI:** 10.48101/ujms.v128.9194

**Published:** 2023-03-27

**Authors:** Sofia Vikman, Anders Larsson, Måns Thulin, Torbjörn Karlsson

**Affiliations:** a Department of Medical Sciences, Uppsala University, Uppsala, Sweden; b Department of Haematology, Uppsala University Hospital, Uppsala, Sweden; c Department of Clinical Chemistry, Uppsala University Hospital, Uppsala, Sweden; d Department of Mathematics, Uppsala University, Uppsala, Sweden

**Keywords:** Essential thrombocythemia, angiogenesis, extracellular matrix, matrix metallopeptidase 9, endostatin

## Abstract

**Background:**

Increased local angiogenesis is important for the growth and dissemination of cancer. The myeloproliferative neoplasm essential thrombocythemia (ET) is known to involve increased bone marrow angiogenesis. Blood levels of several angiogenesis-related proteins are increased in different types of cancer. The aim of this study was to investigate whether a subset of such proteins was elevated in treatment-naïve ET patients.

**Methods:**

Blood plasma from 41 ET patients and 43 healthy aged-matched controls was analyzed for eight different angiogenesis-related proteins.

**Results:**

The ET cohort displayed a more homogenous expression pattern of these proteins compared with controls. Five of the eight proteins were significantly increased in ET patients.

**Conclusion:**

Increased plasma levels of matrix metallopeptidase 9 (MMP9) and endostatin have not previously been reported in ET. In our patients, MMP9 levels correlated positively with Janus kinase 2 (JAK2) V617F allele burden and leukocyte count.

## Introduction

Essential thrombocythemia (ET) is a chronic myeloproliferative neoplasm characterized by clonal megakaryocyte proliferation and thrombocytosis and is associated with hemorrhagic and thrombotic complications (1). Other features of the disease are leukocytosis, splenomegaly, and risk of fibrotic or leukemic transformation. Approximately 60% of ET patients are positive for the Janus kinase 2 (JAK2) V617F mutation (1), which leads to a gain-of-function of this non-receptor tyrosine kinase. Hematopoietic cells that harbor the JAK2 V617F mutation have a proliferative advantage over non-mutated cells. A smaller fraction of ET patients has mutations in the calreticulin (CALR) or MPL proto-oncogene, thrombopoietin receptor (MPL) genes. Only 10–20% of them harbor none of these three driver mutations (1) this group is referred to as having triple negative ET. Angiogenesis is increased in several types of cancer, including ET, where increased bone marrow angiogenesis is observed (2).

Angiogenesis is of importance for tumor progression and the process has been well characterized at the molecular level. Vascular endothelial growth factor (VEGF) is a family of proteins, which regulates angiogenesis and lymphatic angiogenesis. VEGF exerts its effect on angiogenesis by binding and activating specific transmembrane receptor tyrosine kinases (VEGFR) predominantly expressed on endothelial cells (3). The VEGF-VEGFR interaction stimulates proliferation and migration of endothelial cells. Three different VEGF receptors (R1, R2 and R3) have been identified; activation of VEGFR2 by VEGF-A is the most important ligand–receptor interaction for angiogenesis. Proliferation and migration of lymphatic endothelial cells depend on activation of VEGF receptors 2 and 3 by the growth factors VEGF-C and -D (4). Alternative splicing of VEGF and VEGFR pre-mRNA gives rise to different VEGF- and VEGFR protein isoforms, which can have opposing functions in vessel formation (5).

Extracellular matrix (ECM) is the non-cellular tissue component and is mainly composed of proteins such as collagen, polysaccharides and water. The ECM provides structural tissue support as well as exerting a variety of biochemical functions. In cancer there is a disturbance in ECM metabolism, which plays an important role in the creation of a tumor microenvironment (6). Tumor cells promote degradation of ECM, which stimulates angiogenesis via the release of cytokines such as VEGF sequestered in the ECM. Local tumor expansion is also dependent on proteolytic degradation of the surrounding ECM by matrix metallopeptidases (also known as metalloproteinases) (7).

Besides the different VEGF proteins and their receptors and matrix metallopeptidases (MMPs), other proteins such as endostatin, growth differentiation factor 15 (GDF15), pentraxin 3 (PTX3) and selectins are involved in angiogenesis, both in health and disease.

In this study, we investigated plasma levels of eight angiogenesis-related proteins in an attempt to reveal their possible roles in angiogenesis in ET. Plasma levels of endostatin, GDF15, matrix metallopeptidase 9 (MMP9), PTX3, endothelial selectin (E-selectin), platelet selectin (P-selectin) and soluble VEGF receptors (sVEGFR) 1 and 2 were determined by ELISA and compared between ET patients and healthy controls.

## Patients and methods

Written informed consent was obtained from all study participants. The study was approved by the Research Ethics Committee of Uppsala (Refs: 2010/98, 2014/233, Ups-01367, and 2021-03316).

Blood plasma from 41 newly diagnosed treatment-naïve ET patients (32% male) was used for ELISA-analyses of endostatin, E-selectin, P-selectin, GDF15, MMP9, PTX3, sVEGF receptors and C-reactive protein (CRP). Plasma was generated from whole blood left for 30 min before centrifugation at 2,400 g for 7 min at room temperature. EDTA was used as anticoagulant. Time from blood sampling to storage was less than 4 h. Samples were stored in the Uppsala-Umeå Cancer Consortium (U-CAN) Biobank at −80°C before analysis. Laboratory and clinical data and pathology reports from the time of ET diagnosis were obtained from each patient’s individual chart. The Sysmex XN-9000 cell counter (Kobe, Japan) was used to obtain complete blood count. The control group consisted of 43 age- and sex-matched healthy individuals (37% male).

ZZStatistical analyses were calculated using version R 3.6.3 of the R software package (R Foundation for Statistical Computing, Vienna, Austria). Bootstrap t-tests were used to assess the relative differences in biomarker levels between groups (8). Correlations were computed and tested using the Spearman rank correlation. Throughout, *P*-values were adjusted for multiplicity using the Benjamini–Hochberg procedure (9).

## Results

**Figure 1:**
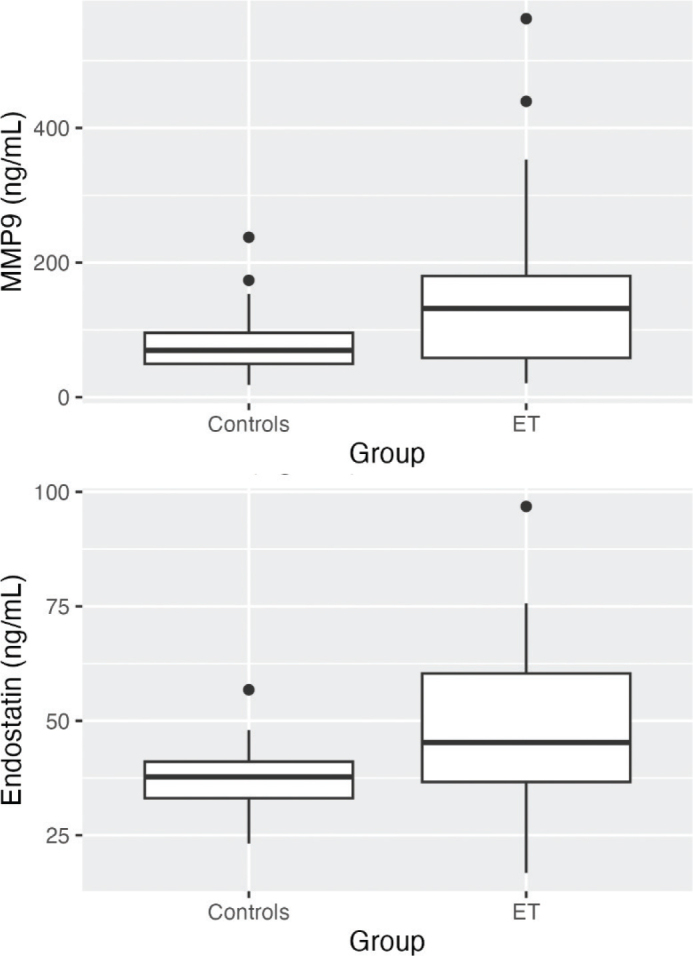
Boxplots visualizing plasma levels of (a) MMP9 and (b) endostatin in ng/mL in controls and ET patients. Medians are shown as thick lines, bottom and top boxes represent the first and third quartiles, whiskers show the smallest and highest non-outliers, and circles represent outliers. In (a) MMP9 in ET > Controls (*P *< 0.01) and in (b) Endostatin in ET > Controls (*P *< 0.001). Abbreviations: ET, essential thrombocythemia; MMP9, matrix metallopeptidase 9.

We analyzed blood plasma from 41 newly diagnosed treatment-naïve ET patients and 43 controls, for levels of CRP, endostatin, GDF15, MMP9, PTX3, E-selectin, P-selectin, sVEGFR1 and sVEGFR2. Median (range) age was 60.5 (25–93) for ET patients and 60 (43–79) for controls. Prior to the ET diagnosis, 33% (13/41) of the patients had been diagnosed with arterial or venous thromboembolic disease. The JAK2 V617F mutation was found in 73% (30/41) of the ET patients, with a median (range) allele burden of 22% (0.13–49.7%) A CALR mutation was found in 10% (4/41) of the ET patients, and none of the group was MPL positive. Clinical and laboratory characteristics of the ET patients and controls are summarized in Table 1. Leukocyte and platelet counts were significantly higher in the ET group (*P *< 0.001 in both cases), whereas mean CRP did not differ between ET patients and controls. Mean endostatin (*P *< 0.001), GDF15 (*P *< 0.001), MMP9 (*P *< 0.01), E-selectin (*P *< 0.05) and P-selectin (*P *< 0.001) plasma levels were higher in the ET group (Table 1, Figure 1). No differences in PXT3, sVEGFR1, or SVEGFR2 were observed between the two groups (Table 1).

**Table 1:** Clinical characteristics and laboratory data for the control and ET populations.

Variable	Controls	ET	*P*
Age (years)^a^	60 (43–79)	60.5 (25–93)	NS
JAK2 V617F allele burden+ (%)^a^	N.A	22 (0.13–49.7)	NA
Hb (g/L)^b^	140 (11)	141 (15)	NS
Leukocytes(×10^9^/L)^b^	6.4 (1.6)	7.9 (3.5)	<0.001
Platelets (×10^9^/L)^b^	251 (80)	587 (284)	<0.001
CRP (mg/L)^b^	1.0 (1.7)	1.5 (2.8)	NS
GDF15 (ng/mL)^b^	521 (204)	720 (737)	<0.001
PTX3 (ng/mL)^b^	1,432 (971)	1,850 (1,385)	NS
E-selectin (ng/mL)^b^	9,618 (4,823)	11,450 (6,936)	<0.05
P-selectin (ng/mL)^b^	44,058 (15,388)	135,959 (62,207)	<0.001
sVEGFR1 (ng/mL)^b^	632 (437)	564 (596)	NS
sVEGFR2 (ng/mL)^b^	3,002 (1,105)	2,891 (1,049)	NS

Variables are expressed as

a median (range) or

b median (interquartile range).

ET: essential thrombocythemia; NA: not applicable; NS: not significant; JAKV617F allele burden+: proportion of positive for Janus kinase 2 allele showing the V617F mutation; CRP: C-reactive protein; GDF15: growth differentiation factor 15; sVEGFR1: soluble vascular endothelial growth factor receptor 1; sVEGFR2: soluble vascular endothelial growth factor receptor 2.

**Figure 2:**
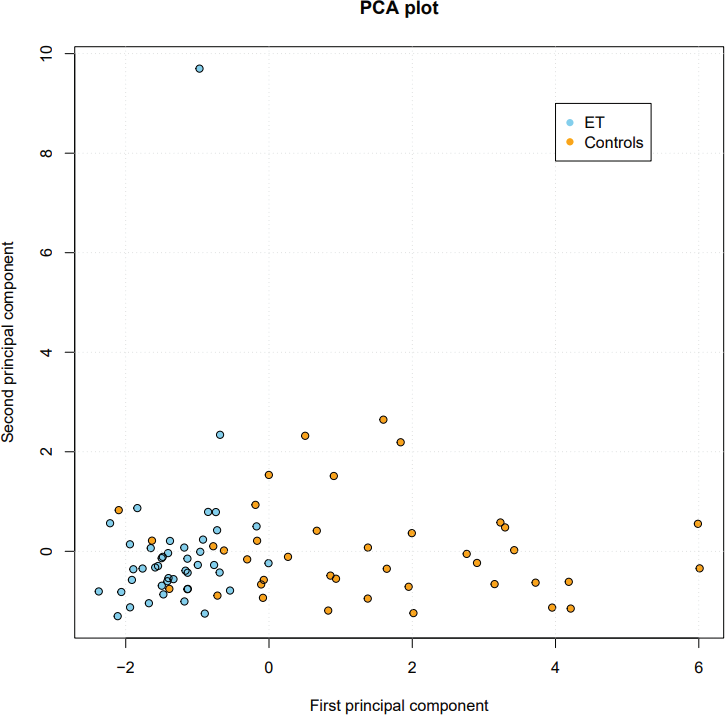
Principal component analysis plot showing homogenous expression of the eight angiogenesis-related proteins analyzed in essential thrombocythemia (ET) patients compared with controls. Controls, yellow dots. ET, blue dots.

A principal component analysis revealed that the ET cohort expressed the proteins more homogenously than did the controls (Figure 2).

Plasma MMP9 correlated positively and significantly with JAK2 V617F allele burden (*P *< 0.05) and leukocyte count (*P *< 0.001) but not with platelet count (Table 2).

**Table 2:** Correlation coefficients (Spearman rank order correlation) of MMP9 with leukocyte and platelet counts and JAK V617F allele burden.

Variable	rs (variable vs. MMP9)	*P*
Leukocyte count	0.68	<0.001
JAK2 V617F	0.42	<0.05
Platelet count	0.11	NS

MMP9: matrix metallopeptidase 9; JAK2 V617F: ratio (%) between V617F mutant and wild type cells (allele burden); NS: not significant.

## Discussion

In an attempt to reveal whether a subset of angiogenesis-related proteins might play a role in the increased bone marrow angiogenesis observed in ET, we compared plasma levels of eight angiogenesis-related proteins between ET patients and controls. As expected, the platelet count was higher among ET patients. The ET group also showed a significantly higher leukocyte count in comparison to controls. Leukocytosis is a common feature in ET (1). Five of the proteins were significantly increased in the ET patients: endostatin, GDF15, MMP9, and E-selectin and P-selectin.

Increased levels of circulating MMPs are observed in several types of cancer (10) and their activity enhances angiogenesis via ECM degradation. MMP9 is secreted by a variety of cells, including neutrophils and macrophages (10). It degrades several components of the ECM, including collagen XVIII, which is expressed in the basal lamina of vessel walls (11). Endostatin is formed by MMP9 degradation of collagen XVIII (11), and so an elevated level of endostatin reflects increased MMP9 activity (12).

MMP9 is essential for formation of new blood vessels *in vivo* (13), and we observed significantly higher plasma levels of both MMP9 and endostatin in ET patients compared with controls. Haas and co-workers have previously shown decreased MMP activity to be associated with a reduction in the number of microscopically observable capillary basement membrane breaks (14). Thus, it is conceivable that increased MMP9 activity facilitates vascular sprouting and capillary growth by making the sub-endothelial basement membrane permeable to migrating and proliferating endothelial cells. Endostatin is primarily an anti-angiogenic agent (15), but the net effect on tumor angiogenesis is determined by the balance between anti- and pro-angiogenic factors. The anti-angiogenic effect of endostatin might be counteracted by MMP9-induced release of VEGF sequestered in the ECM (10).

A certain MMP9 gene polymorphism (Gln279Arg) has been associated with ET (16), but to the best of our knowledge, this study is the first that reports increased plasma MMP9 levels in a population consisting exclusively of ET patients. Polymorphisms in the promoter region of the MMP9 gene have been reported to increase gene expression (17, 18), but the Gln279Arg polymorphism observed in ET is not caused by a mutation in the MMP9 gene promoter. This polymorphism affects MMP9 substrate binding (16), and it is unlikely to increase MMP9 gene expression.

Since neutrophils are the main source of secreted MMP9 in cancer (19), we believe that the increased plasma MMP9 in ET is related to the apparent leukocytosis observed in our patients. Absolute neutrophil count was analyzed in only a few of the patients in our ET cohort, but it is known that leukocytosis in myeloproliferative neoplasms is attributed to neutrophilia (20). This notion is supported by the finding of a positive correlation between MMP9 and leukocyte count. Leukocytes and megakaryocytes stem from a common myeloid progenitor cell (21). Thus, it is likely that the leukocytosis is a result of clonal myelopoiesis rather than cancer-associated inflammation since there was no difference in mean CRP between ET patients and controls. Interestingly, Petzold and co-workers have recently published data that show a role for neutrophils in mobilization of platelets from megakaryocytes (22). Neutrophil-dependent thrombopoiesis requires a direct physical contact between neutrophils and paravascular bone marrow megakaryocytes. This cell-to-cell contact is dependent on binding of the chemokine-receptor CXCR4 to its ligand CXCL12, and so it is conceivable that neutrophilia can exacerbate thrombocytosis in ET.

GDF15, a member of the transforming growth factor β superfamily, is a pro-angiogenic protein. It is abundantly expressed in the placenta during physiological conditions and secreted by activated macrophages as a response to cellular stress signals, such as inflammation, tissue injury and hypoxia (23); its effect is to stimulate proliferation and migration of endothelial colony-forming cells (24). GDF15 exerts its pro-angiogenic effects at least partly by stimulation of VEGF-A synthesis (25). Increased levels of circulating GDF15 are seen in patients with various types of cancer including myeloproliferative neoplasms and in multiple myeloma increased GDF15 is associated with poor prognosis (26, 27). Our current finding of increased plasma GDF15 in ET suggests a possible role for this protein in ET-related increased bone marrow angiogenesis.

Selectins are glycoproteins that are important in immune and inflammatory responses (28). These glycoproteins mediate cell–cell contacts by interaction with their receptors, for example, binding of leukocytes to endothelial cells. Whereas P-selectin is expressed on the surface of platelets and endothelial cells, E-selectin is mainly expressed in endothelium. P- and E-selectin are both involved in angiogenesis (29, 30). P-selectin is increased in ET (31, 32), but there is conflicting evidence regarding E-selectin and ET. Our data showing increased plasma E-selectin in ET are in contrast to the results of Bilgir and co-workers but in line with the finding of others (31–33).

Soluble VEGF receptor 1 is commonly regarded as a decoy receptor for VEGF-A, hence acting as an anti-angiogeneic agent. For example, sVEGFR1 is known to maintain corneal avascularity (34), but there are published data indicating that it is required for normal vascular development (35). Lymphangiogenesis, which is an important factor in tumor progression, is induced by VEGF-C binding its transmembrane receptor VEGFR3. The receptor VEGFR2 exists in a soluble form, which is a result of alterative splicing. Soluble VEGFR2 binds VEGF-C, thus inhibiting VEGF-C/VEGFR3 mediated signaling, which leads to decreased lymphatic endothelial cell proliferation (4). We did not observe any difference in plasma levels of sVEGFR1 when comparing ET and controls. This is in contrast to other reports on sVEGFR1 in myeloproliferative neoplasms (36). The reason for these conflicting data is currently unknown.

Serum VEGF-A is increased in ET (37), which could be relevant given its potential involvement in an alternative way of vessel endothelialization, that is not dependent on local endothelial cell migration and proliferation. This process involves VEGF-dependent recruitment of circulating CD34^+^/VEGFR2^+^ endothelial precursor cells into growing capillaries, where they differentiate into mature endothelium (38). VEGF-A binds to and activates VEGFR2 expressed on endothelial cells, and so decreased sVEGFR2 could possibly stimulate migration and differentiation of CD34^+^/VEGFR^+^ endothelial precursor cells via an increased VEGF-A effect on these cells. The mechanism behind this effect would be less circulating sVEGFR2 able to bind and neutralize VEGF-A. In one study sVEGFR2 was decreased in ET (37), but in our ET cohort mean plasma sVEGFR2 did not differ significantly between ET patients and controls.

PTX3 and CRP, both belong to the pentraxin protein superfamily. However, in contrast to the hepatic protein CRP, PTX3 is produced by different cell types including endothelial cells. PTX3 has dual roles in angiogenesis, since it has both anti- and pro-angiogenic properties (39, 40). In this study, mean plasma PTX3 did not differ significantly between the ET and control cohorts.

Principal component analysis of plasma proteins in leukemia and Hodgkin lymphoma has revealed distinct and homogenous expression patterns compared with controls (41, 42). In line with these findings, we here report that the expression pattern of eight angiogenesis-related proteins is more homogenous in ET than in controls. This can at least partly be explained by the clonality of the disease.

An obvious limitation of this explorative study is that it is retrospective and performed at a single centre. In summary, it reveals that a subset of angiogenesis-related proteins is increased in plasma from treatment-naïve ET patients compared with healthy controls and hence that these proteins may be involved in the increased bone marrow angiogenesis observed in this neoplasm. Increased plasma endostatin and MMP9 in ET have not been reported previously, and further studies are necessary to define their possible roles in angiogenesis in myeloproliferative disorders.
